# Effects of voluntary and forced physical exercise on the retinal health of aging Wistar rats

**DOI:** 10.1007/s11357-024-01208-x

**Published:** 2024-05-25

**Authors:** Anna Szilágyi, Barbara Takács, Réka Szekeres, Vera Tarjányi, Dávid Nagy, Dániel Priksz, Mariann Bombicz, Rita Kiss, Adrienn Mónika Szabó, Andrea Lehoczki, Rudolf Gesztelyi, Béla Juhász, Zoltán Szilvássy, Balázs Varga

**Affiliations:** 1 Department of Pharmacology and Pharmacotherapy, Faculty of Medicine, University of Debrecen, Nagyerdei Krt 98., 4032 Debrecen, Hungary; 2 Departments of Hematology and Stem Cell Transplantation, South Pest Central Hospital, National Institute of Hematology and Infectious Diseases, Saint Ladislaus Campus, Budapest, Hungary; 3 Department of Public Health, Semmelweis University, Budapest, Hungary; 4 Doctoral College, Health Sciences Program, Semmelweis University, Budapest, Hungary

**Keywords:** Physical exercise, Aging, Retinal function, Electroretinography, Wistar rat, MAO-B

## Abstract

Aging is accompanied by an increased prevalence of degenerative conditions, including those affecting ocular health, which significantly impact quality of life and increase the burden on healthcare systems. Among these, retinal aging is of particular concern due to its direct link to vision impairment, a leading cause of disability in the elderly. Vision loss in the aging population is associated with heightened risks of cognitive decline, social isolation, and morbidity. This study addresses the critical gap in our understanding of modifiable lifestyle factors, such as physical exercise, that may mitigate retinal aging and its related pathologies. We investigated the effects of different exercise regimens—voluntary (recreational-type) and forced (high-intensity)—on the retinal health of aging Wistar rats (18-month-old), serving as a model for studying the translational potential of exercise interventions in humans. Male Wistar rats were divided into four groups: a young control (3-month-old) for baseline comparison, an aged sedentary control, an aged group engaging in voluntary exercise via a running wheel in their cage, and an aged group subjected to forced exercise on a treadmill for six sessions of 20 min each per week. After a 6-month experimental period, we assessed retinal function via electroretinography (ERG), measured retinal thickness histologically, and analyzed protein expression changes relevant to oxidative stress, inflammation, and anti-aging mechanisms. Our findings reveal that voluntary exercise positively impacts retinal function and morphology, reducing oxidative stress and inflammation markers while enhancing anti-aging protein expression. In contrast, forced exercise showed diminished benefits. These insights underscore the importance of exercise intensity and preference in preserving retinal health during aging. The study highlights the potential of recreational physical activity as a non-invasive strategy to counteract retinal aging, advocating for further research into exercise regimens as preventative therapies for age-related ocular degenerations.

## Introduction

The aging of the global population represents one of the most significant demographic trends of the twenty-first century, with projections indicating that the number of individuals over 65 years will nearly triple by 2050 [1]. This shift is accompanied by an increase in average life expectancy, heralding remarkable achievements in healthcare and living standards [1]. However, it also introduces substantial challenges, particularly in the form of age-related diseases that disproportionately affect the elderly. Among these, ocular conditions such as age-related macular degeneration (AMD), diabetic retinopathy (DR), and glaucoma stand out due to their profound impact on vision and quality of life [2–4]. Vision impairment, which affects more than 20% of those aged 80 and above, not only diminishes quality of life but also significantly elevates the risk of developing dementia, underscoring the critical need for effective interventions[4, 5].

The aging process is intrinsically linked to a myriad of physiological and molecular alterations that predispose individuals to a spectrum of degenerative conditions, including those compromising ocular health [3, 4, 6–8]. The retina, in particular, is susceptible to age-related functional and structural deterioration, which is central to vision loss among the elderly [3, 4, 7]. These changes are largely driven by the cumulative effects of oxidative stress and chronic inflammation, leading to the progressive decline in retinal health [3, 4, 7, 8]. Accumulation of reactive oxygen species (ROS) may occur due to defects in the electron transport chain and increased monoamine oxidase (MAO) enzyme activity leading to insufficient antioxidant system function [9]. The resulting impairment in vision fundamentally alters the ability of an individual to interact with their environment, further exacerbating the challenges associated with aging.

Despite the acknowledged role of oxidative stress and inflammation in retinal aging, the exploration of lifestyle interventions capable of mitigating these detrimental effects remains limited. Physical exercise, a modifiable lifestyle factor with established benefits across various health domains, presents a promising avenue for research [10–14]. Its potential to counteract the functional, structural, and molecular hallmarks of aging [15–18] in the retina and, by extension, preserve visual function, warrants a deeper investigation[14, 19]. The gap in understanding how different exercise regimens influence the health of the aging retina underscores a critical area for scientific inquiry, particularly in light of the growing prevalence of age-related ocular diseases in an aging population.

Physical exercise is widely acknowledged for its broad health benefits, including improved cardiovascular health, neuroprotection, and enhanced metabolic function [13, 15–18, 20–23]. However, the specific impacts of exercise on the aging eye, particularly at the molecular level, are less well understood. Moreover, exercise can be categorized into voluntary (or recreational) and forced (or compulsory) forms, each potentially eliciting distinct physiological responses [24–31]. Voluntary exercise, characterized by self-initiated activity, is thought to induce positive stress responses and adaptive mechanisms. In contrast, forced exercise, often involving externally imposed regimens, might trigger different or even adverse stress responses due to the lack of control over the exercise intensity and duration. A significant gap in the current knowledge exists regarding how these two forms of exercise influence the aging retina. Specifically, it is unclear whether voluntary and forced exercise differentially affect retinal structure and function in the context of aging.

The primary hypothesis of this study posits that physical exercise, both voluntary and forced, may mitigate age-related degenerative changes in the retina. To evaluate this hypothesis, we examined the effects of a 6-month voluntary and forced exercise regimen on the structure and function of the retina, focusing on retinal thickness and electroretinography (ERG) measurements as indicators of ocular health in aged Wistar rats. Additionally, we assessed the impact of these exercise regimens on the expression of the oxidative stress marker MAO-B, the inflammation marker GFAP, and the neuroprotective markers SIRT6 and BDNF, to elucidate the molecular mechanisms by which physical activity may influence retinal aging.

## Methods

### Animals and experimental groups

**Fig. 1:**
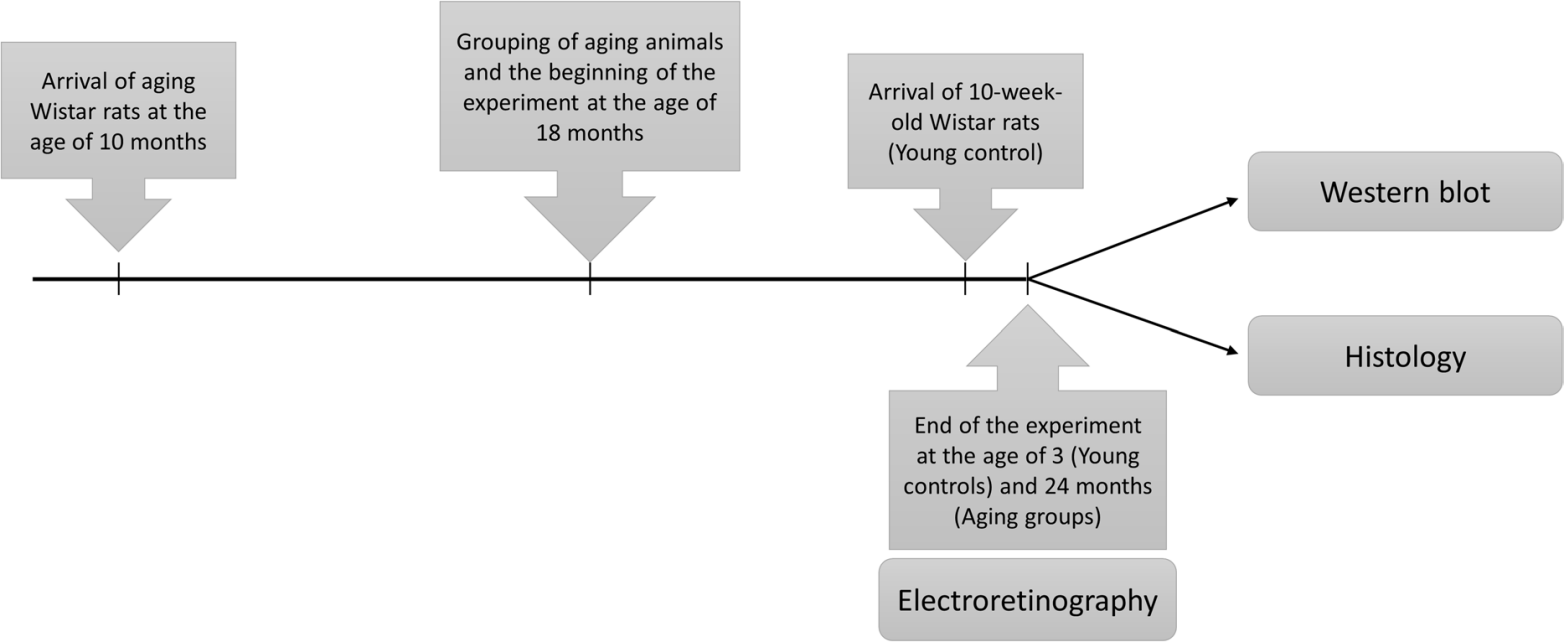
Timeline of the experiment

Male Wistar rats, sourced from Janvier Labs (Rte du Genest, 53,940 Le Genest-Saint-Isle, France), were housed with free access to water and standard rodent chow (Ssniff, Ferdinand-Gabriel-Weg 16, 59,494 Soest, Germany) at a consistent 24 °C, under a reversed 12-h light/dark cycle to align with their nocturnal activity patterns. Acquisition of laboratory animals was carried out in two steps: animals for the old experimental groups were purchased at the age of 10 months, and they were kept in our animal house until they reached 18-month-old age. Young controls were procured at 10 weeks old, 2 weeks of acclimatization was provided, and the end of this period coincided with the end of the experiment. Young controls constituted group I (12 weeks old, no exercise, *n* = 12). Old rats were randomly assigned to one of three groups (*n* = 12 in each group): an aged sedentary control group (group II, 18 months old, no exercise), an aged voluntary exercise group equipped with open running wheels in their cages (group III, 18 months old), and an aged forced exercise group subjected to a structured treadmill regimen in a closed, motorized treadmill (group IV, 18 months old). The study was conducted over 6 months for the old animals. Timeline is shown in Fig. 1. Ethical approval was granted by the Institutional Animal Care Committee of the University of Debrecen (authorization number 3/2022/DEMÁB), ensuring adherence to the ARVO Statement for the Use of Animals in Ophthalmic and Vision Research and NIH guidelines.

### Forced running protocol

The forced running group underwent a progressive training program starting with a 5-min acclimatization to the stationary treadmill. The regimen gradually increased from a speed of 5 m/min for 1 min, with daily increments of 0.5 m/min, until reaching 13 m/min. Subsequently, the duration was increased by 1 min daily until achieving 20 min of continuous running. This routine was maintained 6 days a week, with a rest day on Sundays.

### Electroretinography assessment

Following a 6-month period of specified physical exercise regimens, electroretinographic evaluations were conducted on each animal within the study groups (*n* = 12 per group) to assess retinal function [32]. To facilitate these measurements, general anesthesia was induced using an intramuscular injection of a ketamine/xylazine mixture (100/10 mg/kg) (Calypsol, Gedeon Richter Plc., Budapest, Hungary; CP-Xylazin, Produlab Pharma BV, Raamsdonksveer, The Netherlands), ensuring the animals were fully unconscious to prevent distress and movement. Pupil dilation was achieved through the administration of cyclopentolate-containing eyedrops (Humapent, Teva Ltd., Debrecen, Hungary), essential for accurate ERG recordings.

The ERG procedure was executed using the OcuScience (2764 N. Green Valley Parkway Suite 262 Henderson, NV 89014, USA) HMsERG system (model 2000), a veterinary-specific electroretinograph designed for comprehensive ocular assessments. To capture retinal electrical activity accurately, needle reference electrodes were strategically placed under the skin along the jawline. Concurrently, lens measuring electrodes coated with conductive gel were carefully positioned on the cornea to ensure optimal contact, while a grounding electrode was inserted subdermally at the base of the tail to stabilize the measurement system.

Measurements commenced with scotopic testing following 20 minu of dark adaptation, allowing for the evaluation of retinal response under low-light conditions. Retinal responses were recorded across a range of light intensities (100, 300, 1000, 3000, 10,000, and 25,000 mcds/m^2^) to comprehensively assess function. Subsequently, photopic testing was conducted after a 10-min light adaptation period at 30,000 mcds/m^2^, with data captured at 3000 and 10,000 mcd·s/m^2^ to evaluate retinal performance under brighter conditions.

Data analysis, including the evaluation of wave amplitudes and implicit times, was performed using ERGView version 4.380RV, the proprietary software of the OcuScience HMsERG system. This software facilitated a detailed interpretation of the electrical signals generated by the retina in response to light stimuli.

Upon the completion of the ERG measurements, the animals were humanely euthanized, enabling further post-mortem analyses of their ocular tissues using various methodologies.

### Western Blot analysis

Following the humane euthanization of the animals, eyes were promptly excised and immediately submerged in liquid nitrogen, preserving them for subsequent molecular analyses (*n* = 6 per group). The frozen ocular tissues were pulverized into a fine powder and then homogenized in a buffer solution containing an array of compounds aimed at preserving protein integrity (25 mM Tris, 25 mM NaCl, 1 mM Na-Orthovanadate, 10 mM NaF, 10 mM Na-Pyrophosphate, 10 mM Okadaic acid, 0.5 mM EDTA, 1 mM PMSF protease inhibitor cocktail, and distilled water, all sourced from Sigma-Aldrich-Merck KGaA, Darmstadt, Germany). This mixture was further processed using a T10 Ultra-Turrax disperser (IKA-WERKE, Staufen, Germany) to ensure thorough homogenization. Centrifugation of the homogenate at 2000 rpm for 10 min at 4°C separated the cytosolic and mitochondrial proteins in the supernatant from the nuclear fraction in the pellet. The pellet was then resolved in a homogenization buffer enhanced with Triton X-100 tenside and subjected to a second centrifugation at 14,000 rpm for 10 min at 4°C to extract the nuclear proteins from the supernatant. Further centrifugation of the mixed supernatant at 10,000 rpm for 20 min at 4°C yielded the cytosolic fraction aspirated with the supernatant. Protein concentrations of the nuclear and cytosolic fractions were quantified using a FLUOstar Optima spectrophotometer (BMG Labtech, Ortenberg, Germany) and a BCA assay (QuantiPro BCA Assay Kit, Sigma-Aldrich-Merk KGaA, Darmstadt, Germany). Proteins were prepared with Laemmli sample buffer (Sigma-Aldrich-Merck KGaA, Darmstadt, Germany) and subjected to SDS–Polyacrylamide gel electrophoresis (SDS-PAGE, 12% gel, 25 mA for approximately 220 min). Following electrophoresis, proteins were transferred (with 25 V for 90 min) to a nitrocellulose membrane (GE Healthcare, Darmstadt, Germany), which was subsequently blocked with a 3% BSA solution (Sigma-Aldrich-Merck KGaA, Darmstadt, Germany) and incubated overnight in TBST with primary antibodies targeting specific markers (histone 3, beta-actin, BDNF, MAOB, GFAP, and SIRT6, all procured from Abcam, Cambridge, UK: anti-HistoneH3 recombinant rabbit monoclonal antibody, detecting histone 3 (~ 17 kDa), Cat#ab1791; anti-beta-actin mouse monoclonal anti-body, detecting beta-actin (~ 42 kDa), Cat#ab8226; anti-BDNF rabbit monoclonal anti-body (~ 15 kDa), Cat# ab108319; anti-MAOB monoclonal anti-body (~ 59 kDa), Cat# ab259928; anti-GFAP rabbit polyclonal antibody (~ 48 kDa), Cat#ab7260; anti-SIRT6 rabbit monoclonal anti-body (~ 39 kDa) Cat# ab191385). The membranes were then washed with TBST (3 times for 10 min) and treated with HRP-conjugated secondary antibodies (anti-mouse antibody Cat#A4416; anti-rabbit anti-body Cat#A0545; both procured from Sigma-Aldrich-Merck KGaA, Darmstadt, Germany) to facilitate detection. Visualization of the protein bands was achieved using ECL™ Prime Western Blotting Detection Reagent (Cytiva, Amersham, UK) and captured with a LiCor C-Digit® blot scanner. Image analysis, including normalization to the background and standardization to a housekeeping protein (histone 3 for nuclear or beta-actin for cytosolic proteins), was conducted using ImageJ software (version 1.51, National Institutes of Health, Bethesda, MD, USA). This comprehensive analysis was performed on samples from each experimental group to investigate changes in protein expression linked to the different exercise regimens.

### Histology

Following euthanasia, the eyes of the subjects (*n* = 6 per group) were promptly removed to preserve tissue integrity. Upper section of each eye bulb was marked for consistent orientation during later analysis. The bulbs were then injected with and immersed in a 4% paraformaldehyde (PFA) solution (pH 7.4, comprised of 10 g paraformaldehyde, 50 μL 10 N NaOH, 25 mL 10 × PBS, and 200 ml distilled water) for 24 h, ensuring thorough fixation of the ocular tissues.

Subsequent to fixation, the corneas were excised allowing elimination of paraformaldehyde, followed by an extensive wash in water for one hour. The tissue samples were then preserved in 70% ethanol, preparing them for the next stages of histological processing. This included a series of dehydration steps through increasing concentrations of ethanol (70%, 90%, and finally 100%), after which the samples underwent clearing in xylene and were infiltrated and embedded in paraffin wax using Histowax (Histolab Products AB, Gothenburg, Sweden). The prepared eye tissue blocks were sectioned into 4-μm-thick slices using a microtome, focusing on areas proximal to the optic disk for detailed analysis.

The tissue sections were then rehydrated and subjected to hematoxylin and eosin staining. This process began with a 10-min hematoxylin application (Gill-type, GHS2128, Sigma-Aldrich-Merck KGaA, Darmstadt, Germany), followed by a thorough rinse under tap water until the sections achieved a blue hue. Eosin staining was applied for 5 min to contrast the hematoxylin, highlighting cellular and tissue structures.

For microscopic examination, images of the stained sections were captured focusing on the inferior retina near the optic disk, utilizing a Nikon Eclipse 80i microscope equipped with a DS-Fi3 camera through a 40 × objective lens (Nikon Plan Fluor 40/0.75 DIC M/N2 ¥/0.17 WD 0.66). Image analysis and measurements were conducted using the Nikon NIS-Elements BR software (Version 5.41.00), allowing for precise quantification and morphological assessment of the retinal tissue.

### Statistical analysis

For data analysis, GraphPad Prism software (version 9.0, GraphPad Software Inc., La Jolla, CA, USA) was used. Initially, the Shapiro–Wilk normality test was applied to each dataset to assess the distribution of the data, a crucial step for selecting the appropriate statistical tests. Based on this assessment, datasets conforming to a Gaussian distribution were analyzed using a one-way analysis of variance (ANOVA), a method suited for comparing means across multiple groups. Conversely, for data not adhering to normal distribution, the non-parametric Kruskal–Wallis test was employed. Significance was determined based on *p*-values, with thresholds set to identify varying levels of statistical significance. A *p*-value less than 0.05 was considered indicative of a statistically significant difference between groups. To facilitate understanding and interpretation, significance levels were denoted as follows: * indicates *p* < 0.05, ** for *p* < 0.01, *** for *p* < 0.001, and **** for *p* < 0.0001, marking increasingly strong evidence against the null hypothesis. All data are presented as mean + standard error of the mean (SEM).

## Results

### Electroretinography findings

**Fig. 2:**
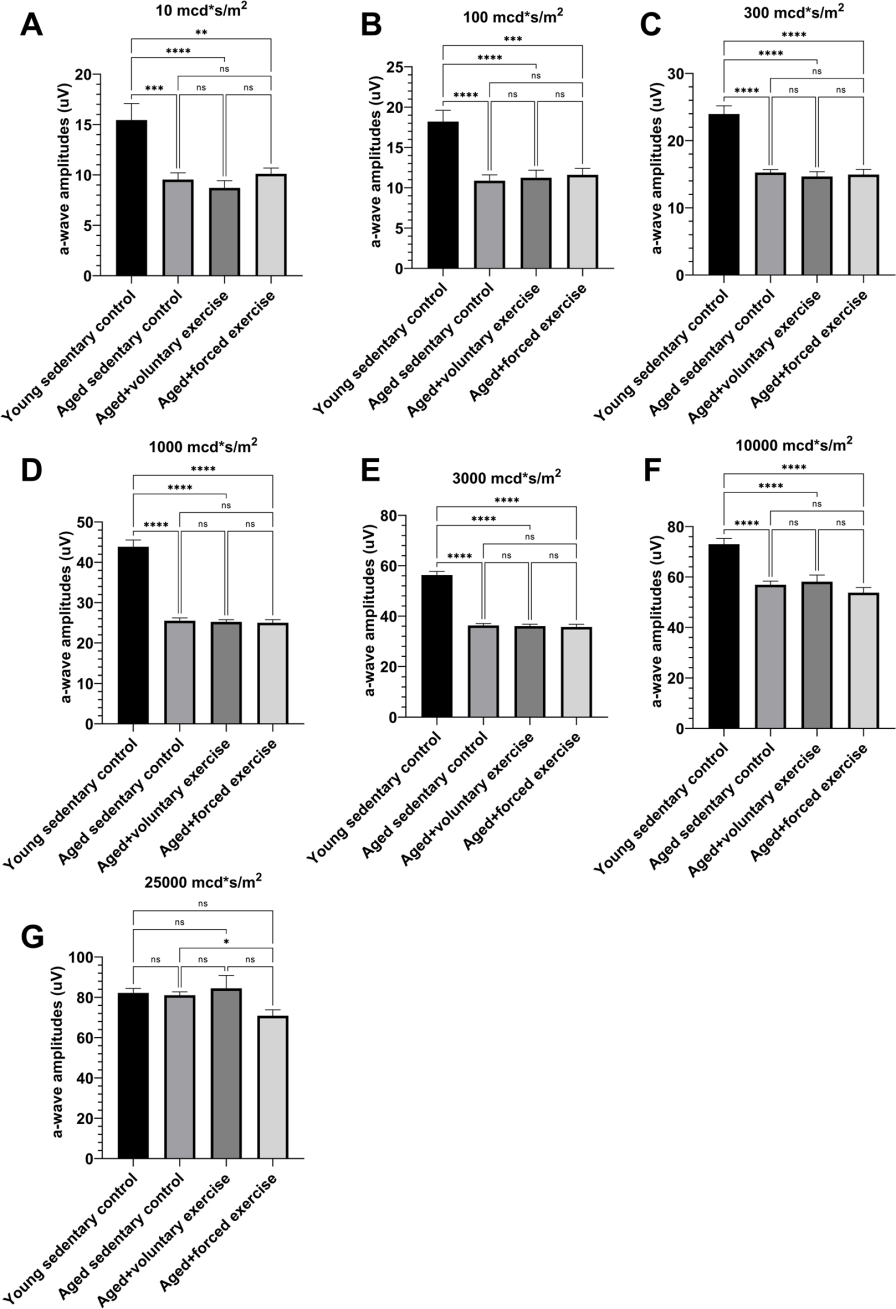
Comparative electroretinography analysis of a-wave amplitudes by light intensity among experimental groups. This figure presents a-wave amplitudes derived from electroretinography (ERG) across a spectrum of light intensities, evaluating the retinal photoreceptor responses of four distinct experimental groups: Young Sedentary Control, Aged Sedentary Control, Aged + Voluntary Exercise, and Aged + Forced Exercise (*n* = 12 in each). The a-wave amplitudes, quantified in microvolts (μV), serve as indicators of electrical reaction of the photoreceptors to visual stimuli. Mean a-wave amplitudes for each group are depicted, with standard error of the mean (SEM) represented by error bars. Statistical analysis outcomes are annotated to reflect significance levels between groups: a *p*-value greater than 0.05 is noted as not significant (ns); * indicates *p* < 0.05; ** denotes *p* < 0.01; *** designates *p* < 0.001; and **** marks *p* < 0.0001. These annotations underscore the comparative photoreceptor activities across different aging and activity conditions at varying light intensities

**Fig. 3:**
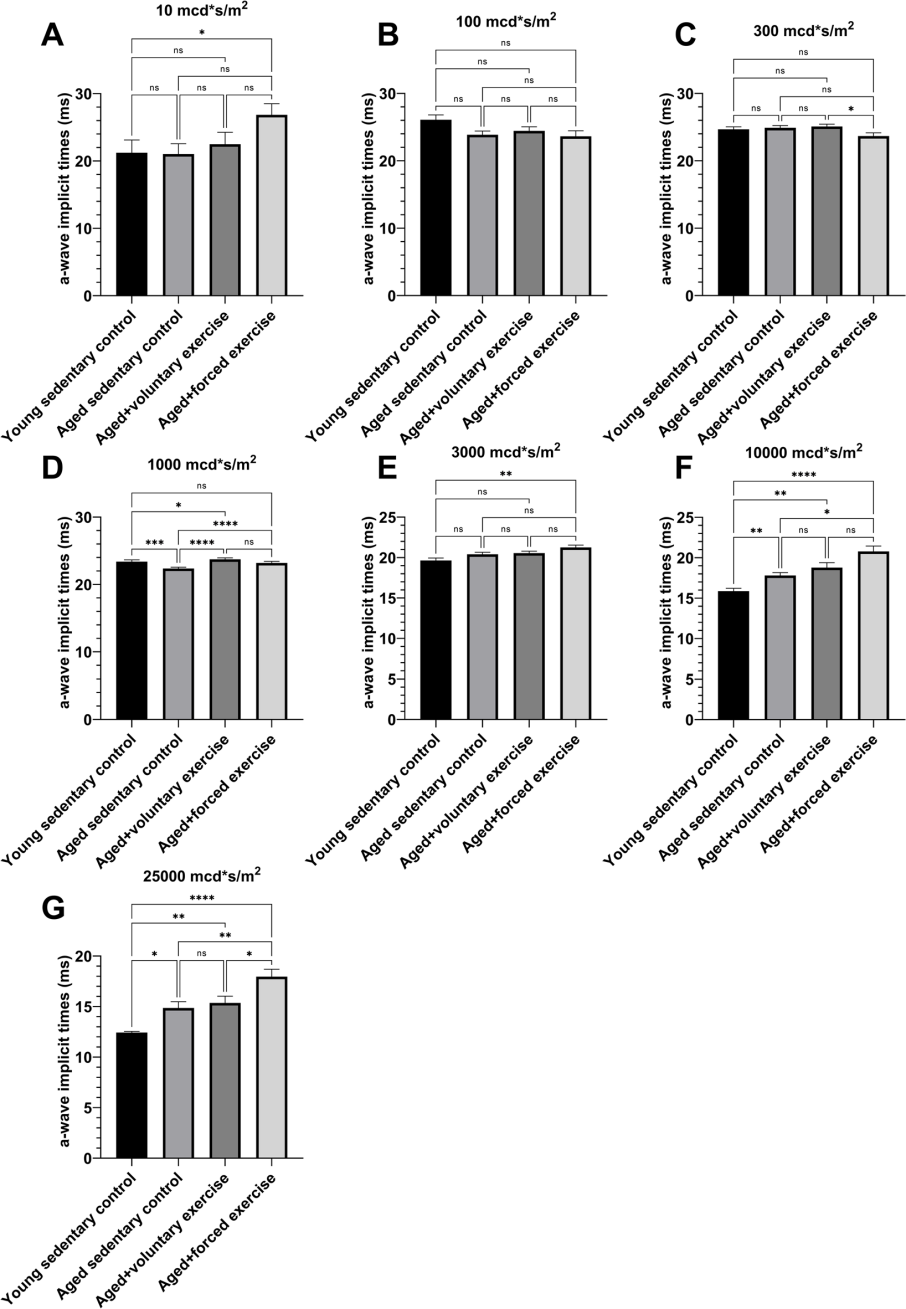
Analysis of a-wave implicit times across light intensities in different experimental groups. This figure displays the implicit times of a-waves recorded through electroretinography (ERG) at varying light intensities, for four experimental groups: Young Sedentary Control, Aged Sedentary Control, Aged + Voluntary Exercise, and Aged + Forced Exercise (*n* = 12 in each). Implicit times, indicating the speed of photoreceptor response to light, are measured in milliseconds (ms) and reflect the temporal dynamics of the retinal response. The graph shows the average implicit times for each group, with error bars representing the standard error of the mean (SEM). Statistical distinctions between the groups are marked as follows: a *p*-value greater than 0.05 is labeled as not significant (ns); * signifies *p* < 0.05; ** denotes *p* < 0.01; *** designates *p* < 0.001; and **** indicates *p* < 0.0001

The Young Sedentary Control group exhibited significantly higher scotopic a-wave amplitudes compared to the older groups. At a high light intensity of 25,000 mcd·s/m^2^, the Aged + Forced Exercise group showed a significant reduction in a-wave amplitudes (Fig. 2). Furthermore, a-wave implicit times were notably prolonged in the Aged + Forced Exercise group, especially at higher light intensities (Fig. 3).

**Fig. 4:**
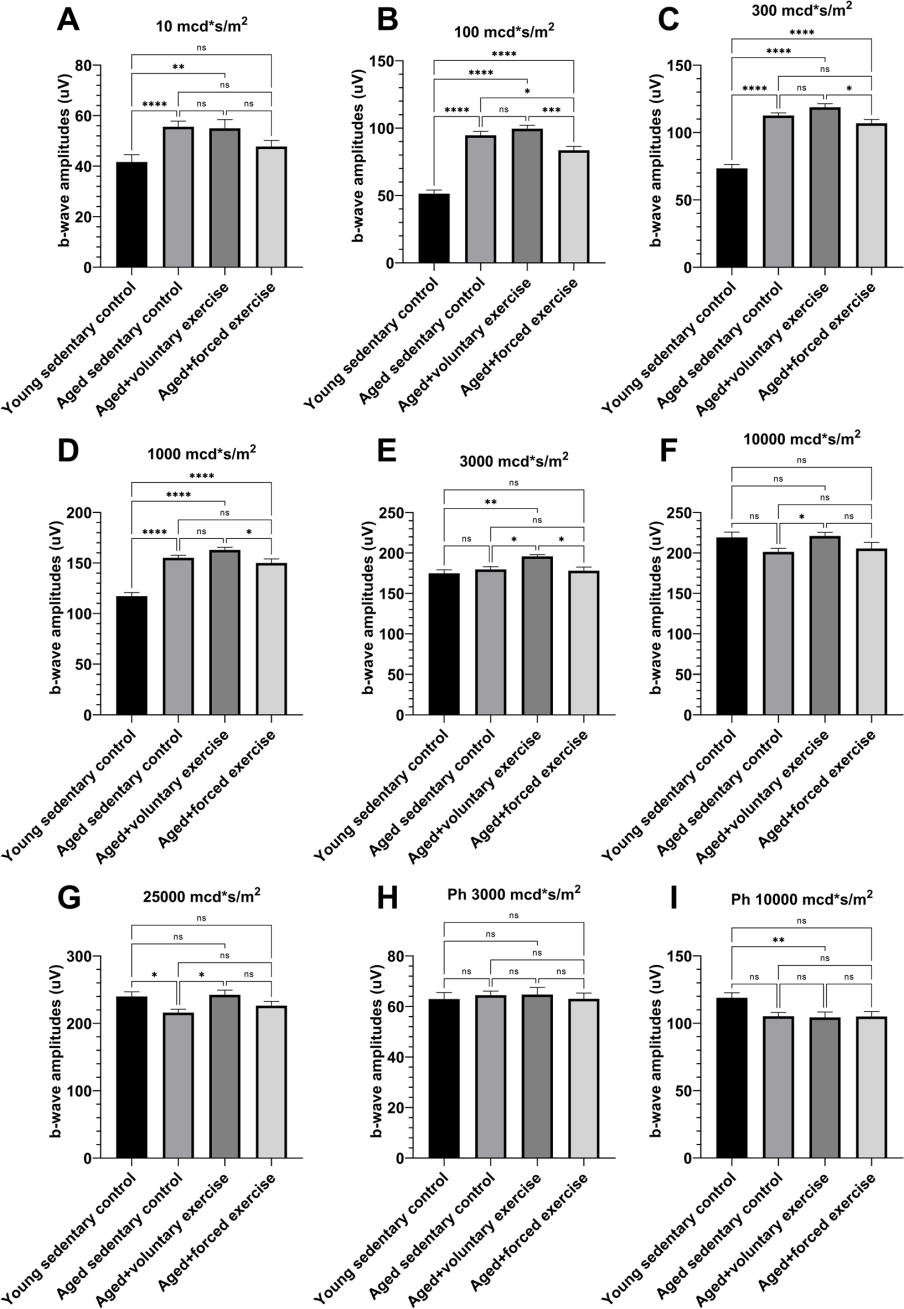
Comparative analysis of b-wave amplitudes by light intensity among experimental groups. This figure showcases the b-wave amplitudes from electroretinography (ERG) measurements conducted at various light intensities for the following groups: Young Sedentary Control, Aged Sedentary Control, Aged + Voluntary Exercise, and Aged + Forced Exercise (*n* = 12 in each). B-wave amplitudes, representing the secondary response of the retina to light, are indicated in microvolts (μV) and serve as a measure of inner retinal activity, particularly from bipolar and Müller cells. The depicted data points illustrate the average b-wave amplitudes for each experimental group, accompanied by error bars denoting the standard error of the mean (SEM). Statistical analysis outcomes between the groups are annotated as follows: not significant (ns) for a *p*-value greater than 0.05; * for *p* < 0.05; ** for *p* < 0.01; *** for *p* < 0.001; and **** for *p* < 0.0001

**Fig. 5:**
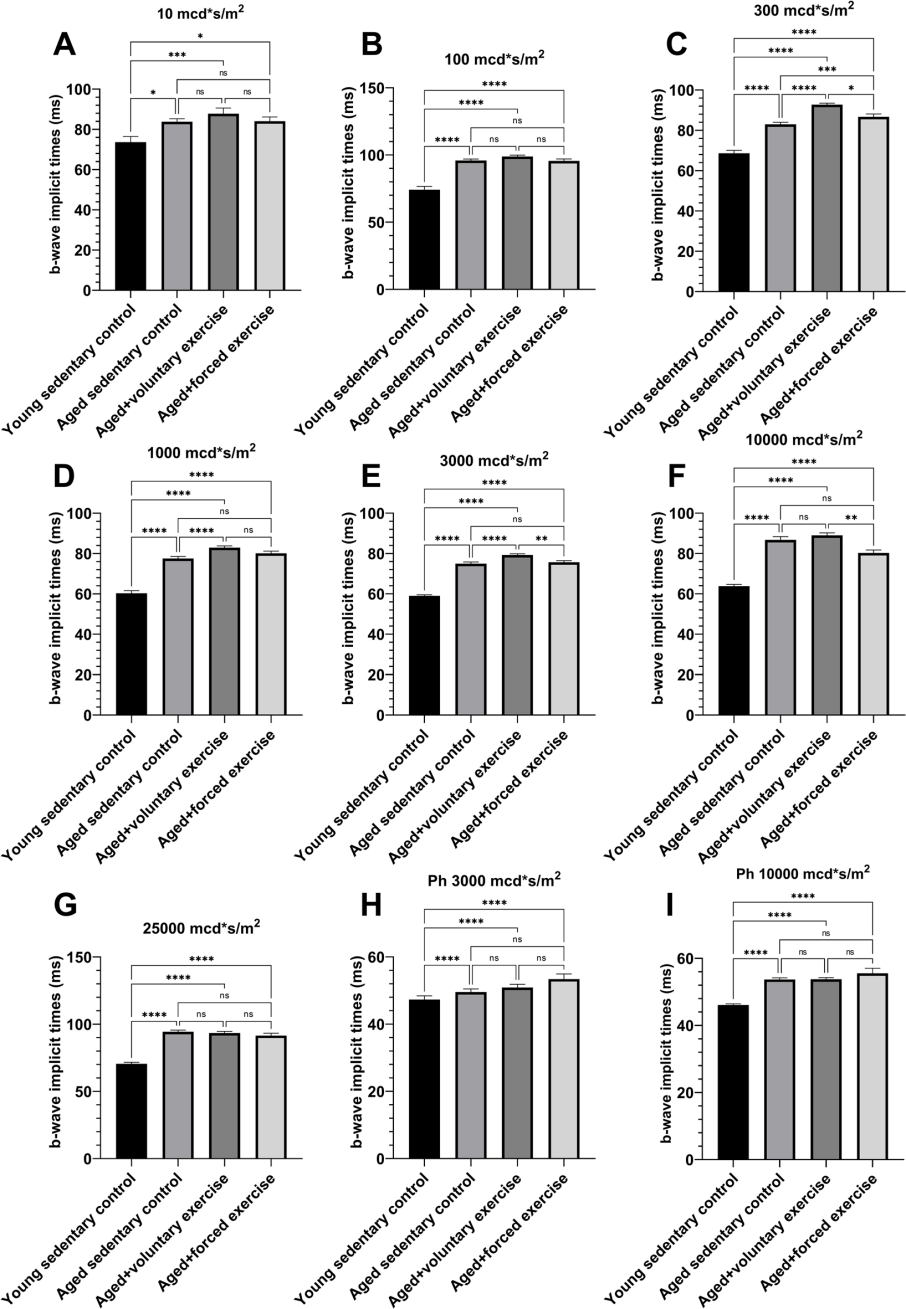
Electroretinography analysis of B-wave implicit times across light intensities for experimental groups. This figure delineates the implicit times of b-waves, as determined through electroretinography (ERG), across a range of light intensities for four distinct groups: Young Sedentary Control, Aged Sedentary Control, Aged + Voluntary Exercise, and Aged + Forced Exercise (*n* = 12 in each). The implicit times, measured in milliseconds (ms), reflect the delay from light stimulus to the peak of the b-wave response, providing insights into the timing of inner retinal signal processing. Displayed data points represent the group mean of b-wave implicit times, with error bars indicating the standard error of the mean (SEM). The statistical significance between the different groups at various light intensities is indicated as follows: not significant (ns) for *p*-values greater than 0.05; * for *p* < 0.05; ** for *p* < 0.01; *** for *p* < 0.001; and **** for *p* < 0.0001. This figure underscores the variations in the speed of retinal processing among the groups under different lighting conditions, shedding light on how age and physical exercise regimes influence retinal signal transmission dynamics

Under low-intensity scotopic conditions, b-wave amplitudes of the Young Sedentary Control group were significantly lower than those of the aged groups. This difference lessened and eventually inverted under high-intensity photopic settings. Among the aged groups, the Aged + Voluntary Exercise group consistently demonstrated higher b-wave amplitudes compared to both the Aged Sedentary Control and Aged + Forced Exercise groups in scotopic measurements (Fig. 4). The Young Sedentary Control group had significantly shorter b-wave implicit times than the older groups (Fig. 5).

### Histological analysis of retinal thickness

**Fig. 6:**
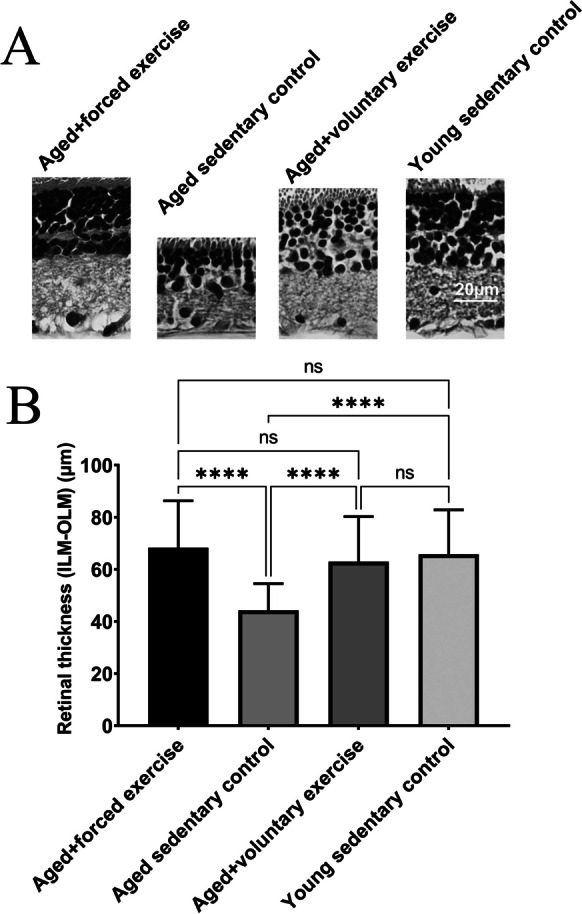
Comparison of retinal thickness across treatment groups. **A** Representative micrographs of the retina for each treatment group, providing visual evidence of structural differences in retinal thickness across the experimental groups: Young Sedentary Control, Aged Sedentary Control, Aged + Voluntary Exercise, and Aged + Forced Exercise (*n* = 6 in each). **B** Bar graphs showing the summary data of retinal thickness among the different treatment groups measured between the inner and outer limiting membrane (ILM, OLM), with error bars representing the standard error of the mean (SEM). Statistical significance between the groups is denoted by: not significant (ns) for *p*-values greater than 0.05 and **** representing *p* < 0.0001. This visualization aims to elucidate the effects of aging and physical activity regimens on retinal structural integrity, highlighting protective benefits of exercise in maintaining retinal thickness in the context of aging

In assessing retinal thickness—specifically, the distance between the inner limiting membrane (membrana limitans interna) and the outer limiting membrane (membrana limitans externa)—our analysis revealed pronounced differences in retinal structural integrity across the studied groups (Fig. 6). The Aged Sedentary Control group exhibited a significantly reduced retinal thickness (44.25 ± 1.813 µm) compared to all other experimental groups, indicating a marked effect of aging on retinal structure (*p* < 0.0001).

However, when comparing the groups engaged in different exercise regimens, no significant differences were observed in retinal thickness. The Young Sedentary Control group showed a retinal thickness of 68.32 ± 3.282 µm, while the Aged + Voluntary Exercise group had a thickness of 62.95 ± 3.056 µm, and the Aged + Forced Exercise group exhibited a thickness of 65.77 ± 3.013 µm. These measurements suggest that both forms of exercise—voluntary and forced—may contribute to mitigating the impact of aging on the retinal structure among the aged populations, maintaining retinal thickness closer to that of younger counterparts (Fig. 6).

### Western blot analysis of protein expression

**Fig. 7:**
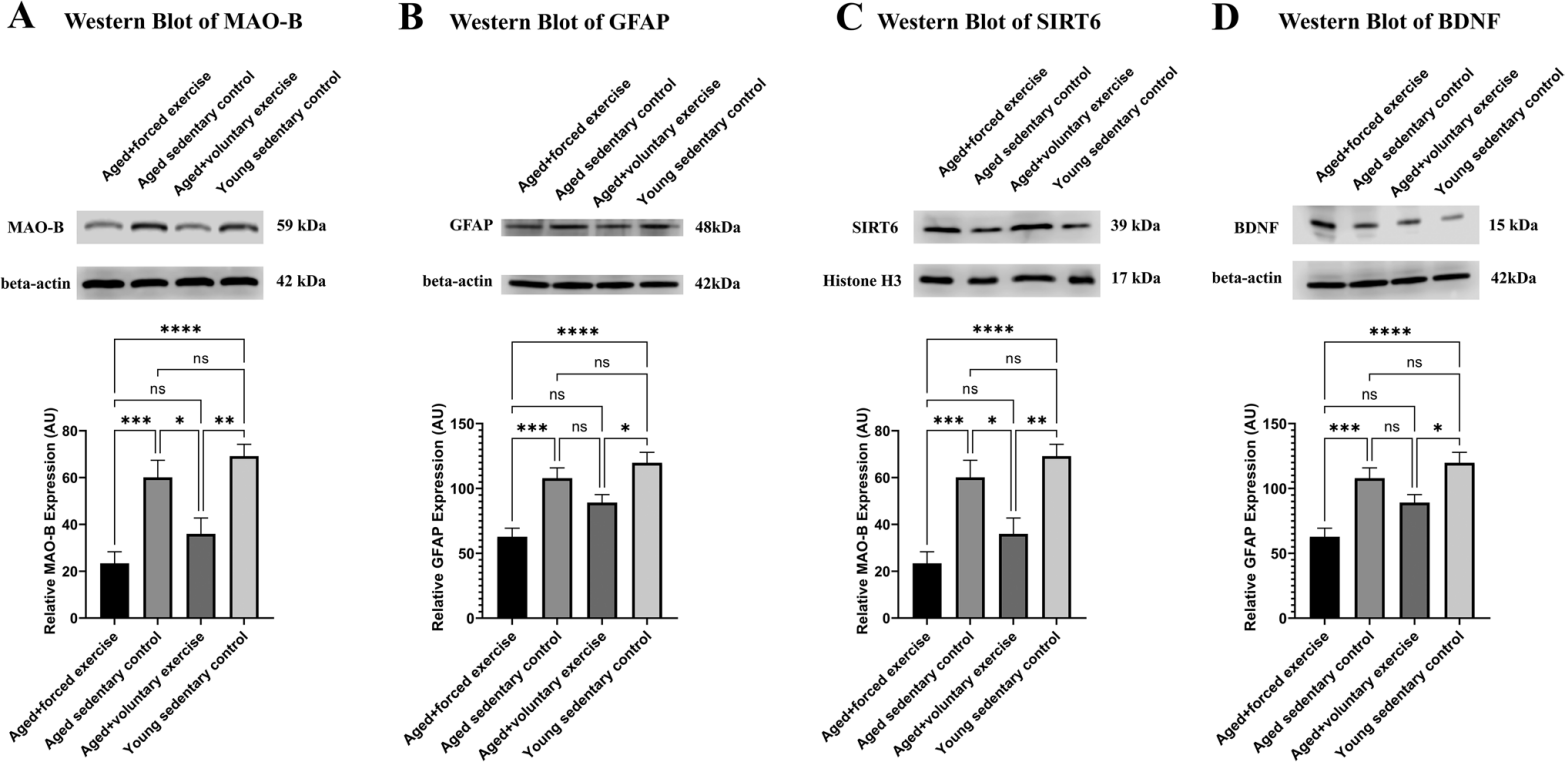
Western blot analysis of protein expression across treatment groups. This figure displays Western blot results showcasing the expression levels of crucial proteins within the retina across various experimental groups: Young Sedentary Control, Aged Sedentary Control, Aged + Voluntary Exercise, and Aged + Forced Exercise (*n* = 6 in each). **A** The expression levels of monoamine-oxidase B (MAO-B) protein, highlighting variations in this oxidative stress marker across the groups. **B** The expression levels of glial fibrillary acidic protein (GFAP), an inflammatory marker. **C** The expression levels of Sirtuin-6 (SIRT6), signaling the activation of anti-aging mechanisms potentially influenced by physical activity. **D** Demonstrates the expression levels of brain-derived neurotrophic factor (BDNF), providing insights into neurotrophic support and neuronal health across the groups. Loading controls were run on separate gels. Results are expressed as mean arbitrary units (AR) with error bars representing the standard error of the mean (SEM) for clear indication of variability. Statistical significance between the groups is marked as follows: not significant (ns) for *p*-values greater than 0.05; * for *p* < 0.05; ** for *p* < 0.01; *** for *p* < 0.001; and **** for *p* < 0.0001

Monoamine oxidase B (MAO-B) is a key enzyme involved in oxidative stress, particularly in the aging brain and eye, where its increased expression can indicate elevated oxidative damage [33–37]. In this context, MAO-B levels were measured to assess the impact of different exercise regimens on oxidative stress within the retinal tissue, a crucial aspect of aging-related ocular health deterioration. We found significantly elevated MAO-B levels in the Aged Sedentary Control and Aged + Forced Exercise groups compared to the Young Sedentary Control and Aged + Voluntary Exercise groups (69.17 ± 5.032 and 60.08 ± 7.314 vs. 23.39 ± 4.938 and 35.98 ± 6.74, respectively; *p* < 0.05). This suggests that forced exercise may not mitigate oxidative stress to the same extent as voluntary exercise or youth (Fig. 7A).

As an indicator of astrocytic activation and neuroinflammation [38–42], GFAP levels were evaluated to explore the effect of exercise on retinal inflammation. GFAP protein levels were notably higher in the Aged Sedentary Control and Aged + Forced Exercise groups compared to the Young Sedentary Control, with the Aged + Voluntary Exercise group showing significantly lower levels than the Aged + Forced Exercise group (107.9 ± 7.929, 119.8 ± 8.034 vs. 62.7 ± 6.644 and 89.05 ± 6.185, respectively), highlighting the potential anti-inflammatory benefits of voluntary exercise (Fig. 7B).

SIRT6 is measured for its association with longevity and resistance to oxidative damage [43, 44]. We observed a significant decrease in SIRT6 levels in the Aged Sedentary Control and Aged + Forced Exercise groups when compared to the Young Sedentary Control, underscoring the challenges of maintaining cellular health in the aging retina. SIRT6 levels of the Aged + Voluntary Exercise group were closer to those of young animals, suggesting a protective effect of voluntary exercise against age-related decline (103.8 ± 6.455, 89.51 ± 5.9 vs. 134.6 ± 10.88 and 122.2 ± 8.278, respectively) (Fig. 7C).

Brain-derived neurotrophic factor (BDNF) is critical for neuronal survival, development, and plasticity [45, 46]. Changes in BDNF expression within the eye could reflect neuroprotective effects of physical activity, making it a relevant marker for studying how exercise influences neurotrophic support in aging ocular tissues. BDNF levels were significantly reduced in the Aged + Forced Exercise group compared to the Young Sedentary Control, indicating potential neurotrophic deficiencies associated with forced exercise in aging. The Aged + Voluntary Exercise group, however, did not show significant differences from the younger or other aged groups, suggesting a nuanced impact of exercise type on neurotrophic support (27.01 ± 4.476 vs. 49.73 ± 6.539 and 38.54 ± 4.392, respectively) (Fig. 7D).

## Discussion

This study sought to investigate the potential protective effects of physical exercise on the aging retina, focusing on both structural and functional aspects, as well as molecular markers related to oxidative stress, inflammation, and neuroprotection. Our findings reveal that both voluntary and forced exercise regimens have distinct impacts on the retinal health of aged Wistar rats, offering insights into the complex interplay between physical activity and ocular aging.

The integrity and functionality of the retina, constantly exposed to environmental stressors such as light, play a crucial role in maintaining visual acuity throughout life. Aging of the retina inevitably leads to a functional decline, characterized by diminished capacity to process visual signals, which is a significant concern for maintaining quality of life in older adults. Light exposure exacerbates this decline, causing cumulative retinal damage that accelerates aging effects and adversely affects neural excitability and synaptic transmission [47]. These age-related alterations in retinal function can be effectively tracked using ERG [48], a non-invasive diagnostic technique that yields detailed insights into the functional status of various retinal cells. In examining the relationship between retinal aging and ERG responses, our findings align with existing literature that documents a decline in ERG performance with age. This decline reflects the functional deterioration inherent to the aging retina, supporting the concept that age-related changes impact the electrical responses of retinal cells to light stimuli. Our study revealed that both voluntary and forced exercise regimens exert distinct influences on ERG measures, with voluntary exercise showing a more pronounced benefit in mitigating the age-related decline in retinal functionality. This suggests that engaging in physical activity, especially when self-selected, may offer protective effects against the functional deterioration observed in the aging retina.

The first negative component on the ERG, the a-wave represents the activity of photoreceptors, which are responsible for the transformation of light to electrical stimuli. Our ERG analysis revealed significant disparities in a-wave amplitudes between young and aged rats, indicating a decrease in either the electrical activity or the number of photoreceptors in the aged groups. This was particularly evident at lower light intensities, predominantly engaging rod photoreceptors, responsible for scotopic vision. The diminishing difference in a-wave responses at higher light intensities, where cone photoreceptors predominate [49, 50], suggests that aging may selectively affect rods more than cones. This presumption aligns with our observation that cone activity in the group of aged rats subjected to forced exercise declined with increasing light intensity, hinting at a possible adverse effect of forced exercise on cone functionality at an old age.

The elongation of a-wave implicit times in the group of aged rats subjected to forced exercise under strong light impulses further points to a decreased sensitivity or delayed response of cones, a condition potentially exacerbated by forced exercise. In retinal physiology, shorter implicit times are indicative of healthier, more efficient photoreceptor function [51], and their prolongation in our study underscores the risk of exercise-induced strain on cone photoreceptors in aging retinas.

The second deflection on the ERG, the positive b-wave, is predominantly the result of currents generated by ON-bipolar cells and other cells localized postsynaptically to the photoreceptors [49, 50]. The b-wave analysis provided additional insights into the retinal impact of aging and exercise. In line with literature amplitudes of scotopic, rod-mediated b-waves of young animals were decreased as these reach maturity slower than mixed rod-cone reactions [52]. Notably, the group of aged rats subjected to voluntary exercise demonstrated superior performance under scotopic conditions compared to other aged groups. This suggests a potential preservation or enhancement of functionality of rod-pathway secondary retinal cells with voluntary exercise. In contrast, the reduced b-wave amplitudes and altered implicit times in the group of aged rats subjected to forced exercise across various light intensities may reflect a detrimental effect on these cells, possibly due to a reduction in their numbers or efficiency in signal transduction. Furthermore, the elongation of b-wave implicit times across all aged groups, when compared to young rats, signifies a latency in signal transduction, becoming more pronounced under lower light intensities. This latency, which diminishes with increased light intensity, suggests its association with secondary retinal cells of the rod-pathway, corroborating with the findings related to a-waves. Specifically, the longer b-wave latencies observed in old groups under low light intensities, where rod-derived stimuli predominate, align with the hypothesis that these latencies may result from diminished rod activity.

Upon comparing b-wave implicit times among the aged groups, distinct patterns emerged. Under scotopic conditions, the implicit times for the group engaged in voluntary exercise were notably longer, yet this difference lessened with increased light intensity and disappeared under photopic conditions. This pattern implies a significant involvement of the rod-pathway and suggests that forced exercise might lead to a reduction in secondary cell numbers within this pathway. The relatively faster b-wave implicit times in the forced exercise group could be interpreted as a result of a streamlined signal processing pathway due to fewer intermediary cells, which might also explain the observed reduction in b-wave amplitudes. These intricate findings highlight the complex relationship between exercise type and retinal cell functionality in aging eyes. The differences observed in b-wave implicit times and amplitudes suggest that while voluntary exercise seems to bolster retinal health, forced exercise may introduce additional stress, particularly affecting the secondary retinal cells of the rod-pathway. This complex interplay underscores the critical need for a nuanced understanding of how distinct exercise regimens influence retinal aging, at both the cellular and functional levels.

A hallmark of aging within the ocular domain is the progressive thinning of the retina, a phenomenon consistently documented in the literature [53–58]. This age-related retinal thinning was also observed in our study, particularly pronounced within the old sedentary control group of Wistar rats. Remarkably, our findings reveal that regular physical activity, whether through voluntary or forced exercise, appears to mitigate this age-associated decline in retinal thickness. The physically active old groups maintained retinal thickness at levels comparable to those of young control rats, suggesting a protective effect of exercise against the structural deteriorations typically observed with aging.

The preservation of retinal thickness and improved electroretinographic responses observed in the exercise groups, notably more pronounced in the voluntary exercise group, support our hypothesis that physical activity can mitigate various facets of age-related retinal degeneration. These outcomes resonate with existing literature that underscores the beneficial impact of regular physical activity on retinal health through mechanisms such as increased production of trophic factors [13], reduced oxidative damage and inflammation [10, 59], improved metabolic function, capillarization, and enhanced blood flow [11, 60–64] within retinal tissues. Notably, the advantages of a voluntary exercise regimen over a forced one may stem from the stress-reducing benefits of self-selected activity levels, potentially counteracting the adverse effects of chronic stress on the retina.

Age-related changes in the retina are governed by several key mechanisms of cellular aging, which collectively contribute to the structural and functional deteriorations observed in ocular diseases [4, 7, 8, 65]. Central to the discussion of how exercise confers these protective benefits is the role of mitochondria in the aging process and specifically in retinal health [59]. Aging is associated with a decline in mitochondrial function across various tissues [8, 66–75], contributing to decreased cellular energy production, increased oxidative stress, and impaired metabolic balance. In the retina, particularly vulnerable to metabolic and oxidative stress due to its high energy demands and exposure to light, mitochondrial dysfunction plays a significant role in degenerative conditions, including AMD [3, 76–78]. Mitochondrial damage and the resulting energy deficit can exacerbate the vulnerability of retinal cells, leading to progressive loss of vision. Evidence suggests that exercise can profoundly impact mitochondrial health, promoting mitochondrial biogenesis, enhancing efficiency in energy production, and reducing oxidative damage through the upregulation of antioxidant defenses [16, 73, 79–84]. These exercise-induced mitochondrial adaptations are thought to improve the metabolic resilience of cells, including those in the retina, thereby offering protection against age-related structural and functional decline. By stimulating these conserved cellular and molecular mechanisms of aging, regular physical activity, especially when self-regulated, may provide a significant protective buffer against retinal degeneration and other age-associated pathologies. Future investigations into the direct effects of exercise on mitochondrial function within the retina could further elucidate the pathways through which physical activity exerts its protective effects, potentially informing therapeutic strategies aimed at preserving ocular health in the aging population.

Oxidative stress, characterized by an imbalance between the production of reactive oxygen species (ROS) and the capacity of the cellular antioxidant defense systems, plays a crucial role in aging [65, 85–87]. It is a significant factor contributing to retinal aging and AMD [65]. Over time, oxidative stress can induce DNA damage, compromising genomic integrity and cellular function, and leading to cellular senescence [65, 87]. This process of aging-induced cellular senescence further exacerbates inflammation through the senescence-associated secretory phenotype (SASP) [87]. There is emerging evidence demonstrating an important role of senescent cells in retinal aging and the pathogenesis of AMD [6, 65]. Additionally, with aging, the efficiency of autophagy, which is vital for removing damaged cellular components, declines [87]. This reduction contributes to the accumulation of cellular debris and dysfunctional organelles [87].

Physical exercise exerts multifaceted effects on these mechanisms of cellular aging [15, 17, 18], offering potential protective benefits for the retina. Exercise has been shown to enhance the cellular antioxidant capacity, reducing oxidative stress and its detrimental effects on cellular components [15, 18, 81, 83, 88–91]. This is particularly relevant for the retina, where photoreceptor cells are highly susceptible to oxidative damage due to their high metabolic activity and exposure to light. The differential expression of MAO-B, GFAP, SIRT6, and BDNF across the groups underscores the molecular basis of exercise-induced retinal protection. The reduction in MAO-B and GFAP levels in the voluntary exercise groups suggests a decrease in oxidative stress [35–37] and inflammation, key contributors to aging-associated retinal damage. This is in line with literature demonstrating the antioxidant and anti-inflammatory effects of exercise [92–95]. Moreover, the elevation of SIRT6 and BDNF levels in the voluntary exercise group could indicate enhanced DNA repair mechanisms and neurotrophic support, contributing to the maintenance of retinal structure and function [96, 97]. These molecular alterations underscore the capacity of physical exercise to mitigate the adverse effects of retinal aging, paralleling the protective benefits it confers across various organ systems [98–101]. Moreover, exercise promotes DNA repair mechanisms, reducing the accumulation of DNA damage and mitigating senescence [15, 17, 18]. This is crucial for retinal health, considering the continuous exposure of retina to environmental stressors capable of inducing oxidative DNA damage. Physical activity also stimulates autophagy, enhancing the clearance of damaged proteins and organelles [15, 17, 18]. By improving autophagy, exercise helps maintain cellular homeostasis in the retina, offering protection against age-related changes and degeneration.

The impact of exercise on oxidative stress, particularly regarding voluntary versus forced exercise, presents a nuanced relationship with intensity playing a pivotal role. While numerous studies advocate for the beneficial effects of physical activity being proportional to its intensity [93–95], recent evidence suggests a more complex picture, especially in elderly populations [102–104]. Specifically, high-intensity, or forced, exercise regimes do not necessarily confer additional benefits over low-intensity, or voluntary, activities and may, in fact, exacerbate oxidative stress [105, 106]. This is due to the enhanced generation of ROS beyond a manageable threshold during intense physical exertion, potentially leading to cellular and molecular damage [105, 106]. In contrast, voluntary exercise, typically self-paced and hence more likely to be moderate in intensity, aligns better with the capacity of the body to manage ROS production, maintaining a balance that supports cellular health and longevity. This distinction underscores the importance of tailoring exercise programs to individual capabilities and preferences, especially in aging populations, to maximize the health benefits of physical activity while minimizing the risk of oxidative stress-induced damage.

The health benefits of exercise are mediated through a complex interplay of systemic and local factors, among which humoral mediators play a crucial role [107]. These include a variety of hormones, growth factors, and cytokines, often referred to as “exercise factors” or “exerkines,” that are released into the circulation in response to physical activity [108–113]. Myokines, a subset of these factors, are produced and released by muscle fibers during physical activity [110, 114]. They play a pivotal role in mediating the beneficial effects of exercise on health, functioning as crucial communication links between muscles and various organs and tissues throughout the body. These exercise-induced proteins have been shown to have systemic effects that extend well beyond the muscles themselves, influencing processes such as inflammation, metabolic regulation, and even brain function [110, 114]. For example, the myokine irisin has been shown to play a crucial role in protecting endothelial cells, thereby enhancing vascular barrier integrity [115]. Similarly, muscle-derived BDNF not only supports neuronal health [116] but is also believed to extend protective effects to the retina. Regarding the retina, the systemic anti-inflammatory and neuroprotective effects of myokines could potentially offer protection against degenerative changes. The exact mechanisms through which myokines and other exerkines might influence retinal health remain an area of active research. However, it is conceivable that they could help mitigate inflammatory processes within the retina, support neuronal survival, and enhance retinal vascular health. Another important mediator is insulin-like growth factor 1 (IGF-1) [107, 117], which is known to support neurogenesis, synaptic plasticity, and vascular health [118–121], potentially contributing to the cognitive and cardiovascular benefits associated with regular exercise. Given the similarities in vascular and neuronal structures between the brain and retina, the benefits of myokines and IGF-1 observed in promoting brain health, including enhancing neurogenesis and cognitive function, suggest promising avenues for exploring their effects on the retina. The investigation into the role of exerkines and IGF-1 in retinal health represents an exciting frontier that could further unravel how physical exercise exerts its widespread benefits, potentially leading to novel therapeutic strategies for retinal diseases based on enhancing the systemic production of beneficial myokines through targeted physical activity regimens.

The potential benefits of exercise-induced molecules, or exerkines, could be further amplified through the use of exercise mimetics [122, 123]. These pharmacological agents, when designed with precise molecular targets and effective formulations, could offer substantial benefits, particularly for individuals who are unable to engage in physical activity due to disabilities. While it is unrealistic to expect that any drug could fully replicate the complex effects of physical exercise, the use of exercise mimetics or polypills may be most advantageous when used in conjunction with whatever physical activity is feasible, thereby enhancing the health benefits derived from exerkines.

Microvascular pathologies play a significant role in the aging of the retina, contributing to the development of AMD and diabetic retinopathy, two of the leading causes of vision impairment and blindness in the elderly population [2]. Optical coherence tomography angiography (OCTA) has provided valuable insights into these changes, revealing alterations in the density and integrity of the aging retinal microvasculature that are associated with disease progression [2, 124, 125]. The retinal microvasculature, like that of the brain, is crucial for maintaining tissue health by supplying necessary nutrients and oxygen while removing metabolic waste. Disruptions in this delicate network can lead to tissue ischemia, inflammation, and subsequent neuronal and photoreceptor cell death, underscoring the importance of microvascular health in retinal function. Furthermore, the intact barrier function of retinal microvascular endothelial cells plays a pivotal role in ocular health, acting as a critical line of defense against the leakage of plasma proteins into neuronal tissue, which can trigger inflammation and contribute to various pathologies within the retina. This barrier is essential for maintaining the homeostasis of the retinal environment and its disruption can exacerbate the pathological processes underlying AMD. There is a growing body of evidence suggesting that exercise exerts a protective effect on microvascular health, paralleling observations made regarding brain health [107, 126–128]. In the brain, physical activity promotes capillarization and improves cerebral blood flow [126–128], which in turn supports neurogenesis, cognitive function, and resilience against neurodegenerative changes. Similar mechanisms are likely at play within the retina, where enhanced microvascular health could support the metabolic demands of the retinal cells and counteract the microvascular deterioration seen in AMD and diabetic reinopathy. The potential for increased capillarization through regular exercise to contribute to the preservation or even increase of retinal thickness is an intriguing hypothesis that merits further investigation. Such changes in the microvascular architecture, potentially detectable by OCTA, could provide a mechanistic explanation for the protective effects of exercise on retinal structure and function observed in our study and others. Regular physical exercise may also enhance the barrier function of retinal microvascular endothelial cells, potentially mitigating the microvascular contributions to retinal aging and disease. Understanding the impact of exercise on retinal microvascular health could open new avenues for preventing or slowing the progression of retinal aging and related diseases. Future research, incorporating detailed assessments of retinal microvascular changes and their relationship to overall retinal health and function, will be crucial in elucidating the full scope of benefits of exercise on the ocular system. This research could also explore whether the protective effects of exercise on the microvasculature of retina can directly contribute to increased retinal thickness.

In conclusion, this study underscores the significant potential of physical exercise as a key lifestyle intervention for promoting healthy aging, particularly in relation to preserving retinal health. By demonstrating that exercise regimens can mitigate age-related changes in the retina, such as retinal thinning and alterations in electroretinographic responses, our findings advocate for the integration of regular physical activity into strategies aimed at maintaining ocular health in the elderly. Importantly, the observation that voluntary exercise may offer more pronounced benefits highlights the value of incorporating self-selected, enjoyable physical activities into daily routines to maximize adherence and health outcomes.

## Data Availability

Datasets are available on request from the authors; however, restrictions may apply, as the datasets presented in this article are the property of the University of Debrecen. Requests to access the datasets should be directed to the authors.
