# Correction to: Effects of voluntary and forced physical exercise on the retinal health of aging Wistar rats

**DOI:** 10.1007/s11357-025-01558-0

**Published:** 2025-02-19

**Authors:** Anna Szilágyi, Barbara Takács, Réka Szekeres, Vera Tarjányi, Dávid Nagy, Dániel Priksz, Mariann Bombicz, Rita Kiss, Adrienn Mónika Szabó, Andrea Lehoczki, Rudolf Gesztelyi, Béla Juhász, Zoltán Szilvássy, Balázs Varga

**Affiliations:** 1 Department of Pharmacology and Pharmacotherapy, Faculty of Medicine, University of Debrecen, Nagyerdei Krt 98., 4032 Debrecen, Hungary; 2 Departments of Hematology and Stem Cell Transplantation, South Pest Central Hospital, National Institute of Hematology and Infectious Diseases, Saint Ladislaus Campus, Budapest, Hungary; 3 Department of Public Health, Semmelweis University, Budapest, Hungary; 4 Doctoral College, Health Sciences Program, Semmelweis University, Budapest, Hungary

**Correction to: GeroScience (2024) 46:4707–4728**

10.1007/s11357-024-01208-x

The original version of this article unfortunately contained errors in Figures 6 and 7.

The incorrect Figures 6 and 7 are shown below.

**Figure:**
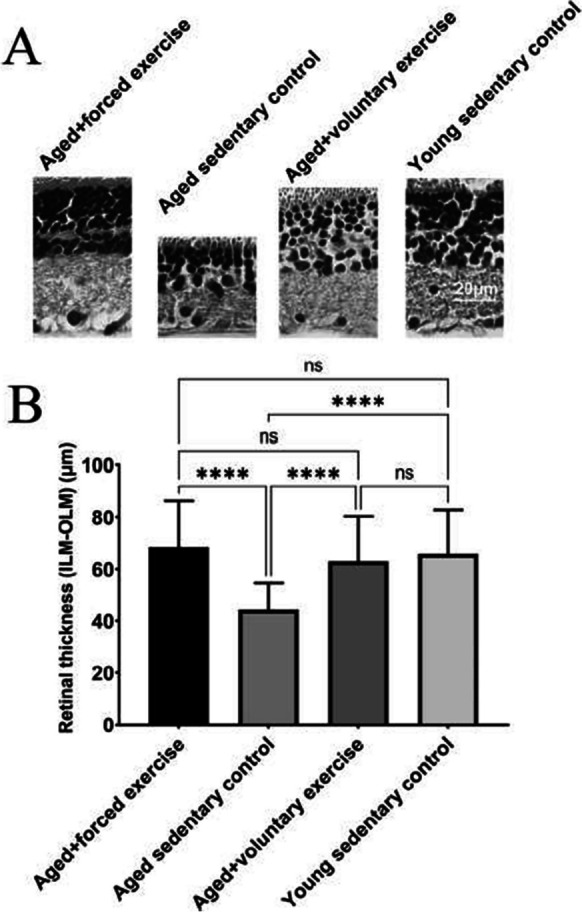
** Fig. 6** Comparison of retinal thickness across treatment groups. A Representative micrographs of the retina for each treatment group, providing visual evidence of structural differences in retinal thickness across the experimental groups: Young Sedentary Control, Aged Sedentary Control, Aged + Voluntary Exercise, and Aged + Forced Exercise ( n = 6 in each). B Bar graphs showing the summary data of retinal thickness among the different treatment groups measured between the inner and outer limiting membrane (ILM, OLM), with error bars representing the standard error of the mean (SEM). Statistical significance between the groups is denoted by: not significant (ns) for p -values greater than 0.05 and **** representing p < 0.0001. This visualization aims to elucidate the effects of aging and physical activity regimens on retinal structural integrity, highlighting protective benefits of exercise in maintaining retinal thickness in the context of aging

**Figure:**
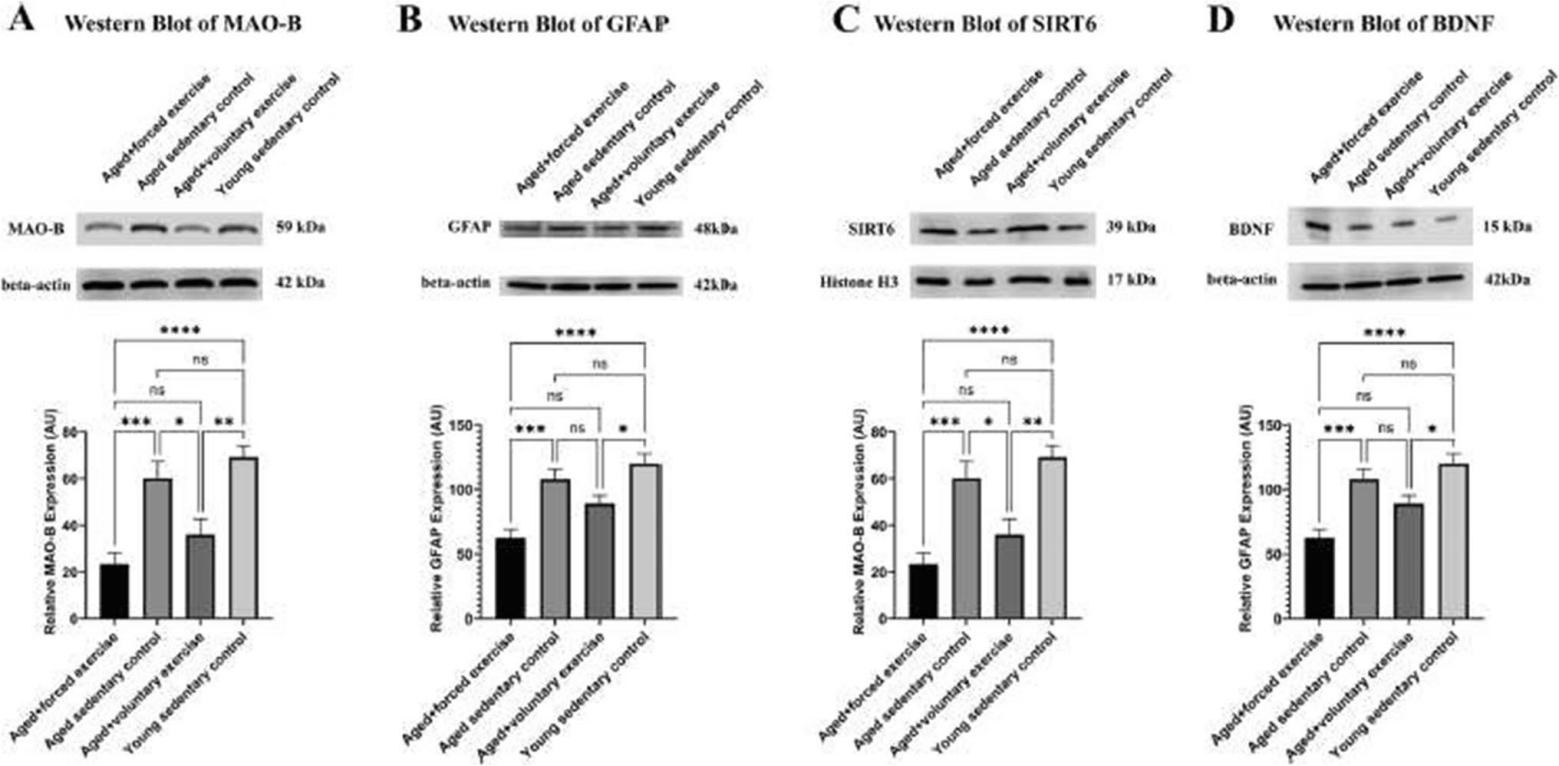
** Fig. 7** Western blot analysis of protein expression across treatment groups. This figure displays Western blot results showcasing the expression levels of crucial proteins within the retina across various experimental groups: Young Sedentary Control, Aged Sedentary Control, Aged + Voluntary Exercise, and Aged + Forced Exercise ( n = 6 in each). A The expression levels of monoamine-oxidase B (MAO-B) protein, highlighting variations in this oxidative stress marker across the groups. B The expression levels of glial fibrillary acidic protein (GFAP), an inflammatory marker. C The expression levels of Sirtuin-6 (SIRT6), signaling the activation of anti-aging mechanisms potentially influenced by physical activity. D Demonstrates the expression levels of brain-derived neurotrophic factor (BDNF), providing insights into neurotrophic support and neuronal health across the groups. Loading controls were run on separate gels. Results are expressed as mean arbitrary units (AR) with error bars representing the standard error of the mean (SEM) for clear indication of variability. Statistical significance between the groups is marked as follows: not significant (ns) for p -values greater than 0.05; * for p < 0.05; ** for p < 0.01; *** for p < 0.001; and **** for p < 0.0001

The corrected Figures 6 and 7 are shown below.

**Figure:**
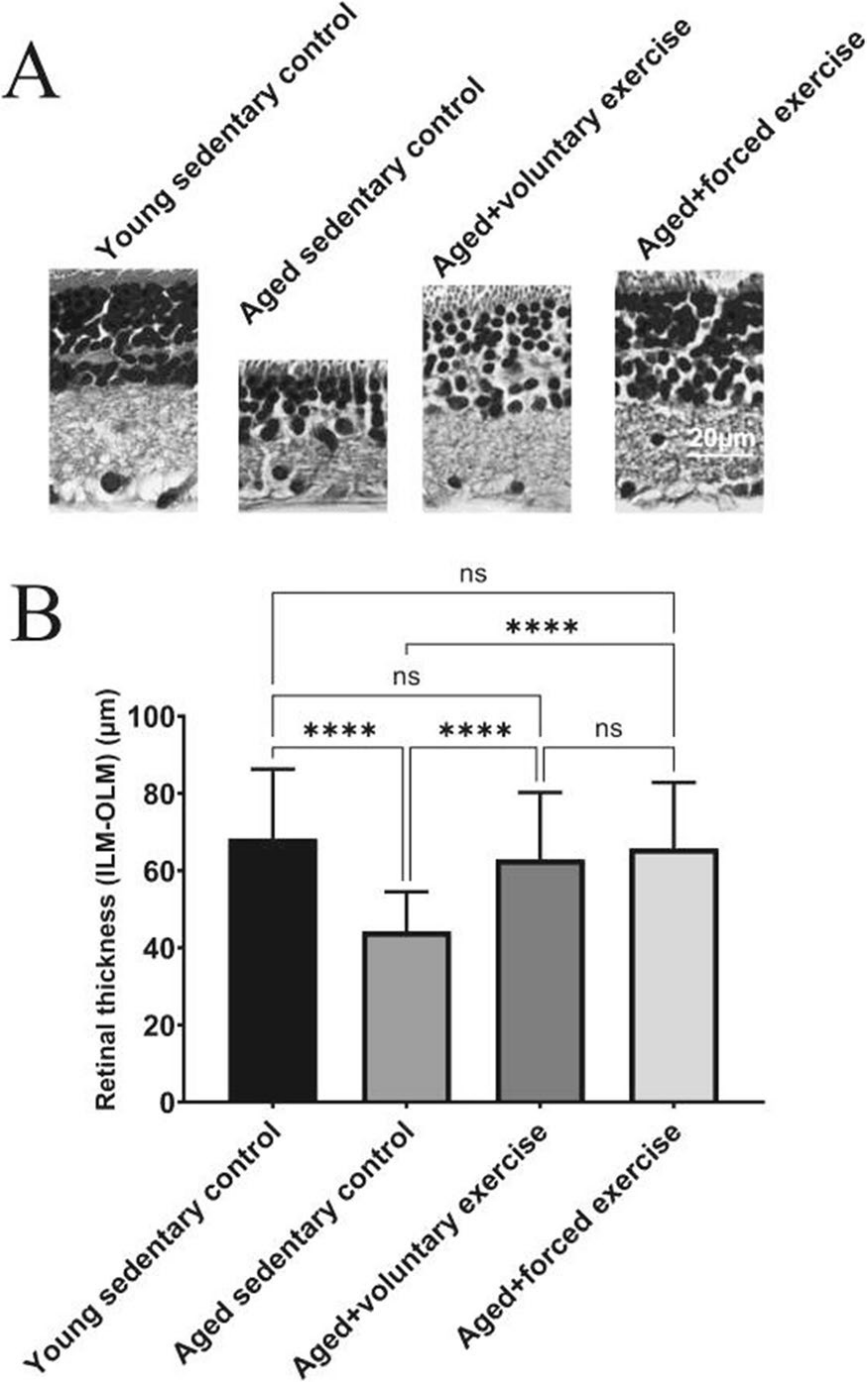
** Fig. 6** Comparison of retinal thickness across treatment groups. A Representative micrographs of the retina for each treatment group, providing visual evidence of structural differences in retinal thickness across the experimental groups: Young Sedentary Control, Aged Sedentary Control, Aged + Voluntary Exercise, and Aged + Forced Exercise ( n = 6 in each). B Bar graphs showing the summary data of retinal thickness among the different treatment groups measured between the inner and outer limiting membrane (ILM, OLM), with error bars representing the standard error of the mean (SEM). Statistical significance between the groups is denoted by: not significant (ns) for p -values greater than 0.05 and **** representing p < 0.0001. This visualization aims to elucidate the effects of aging and physical activity regimens on retinal structural integrity, highlighting protective benefits of exercise in maintaining retinal thickness in the context of aging

**Figure:**
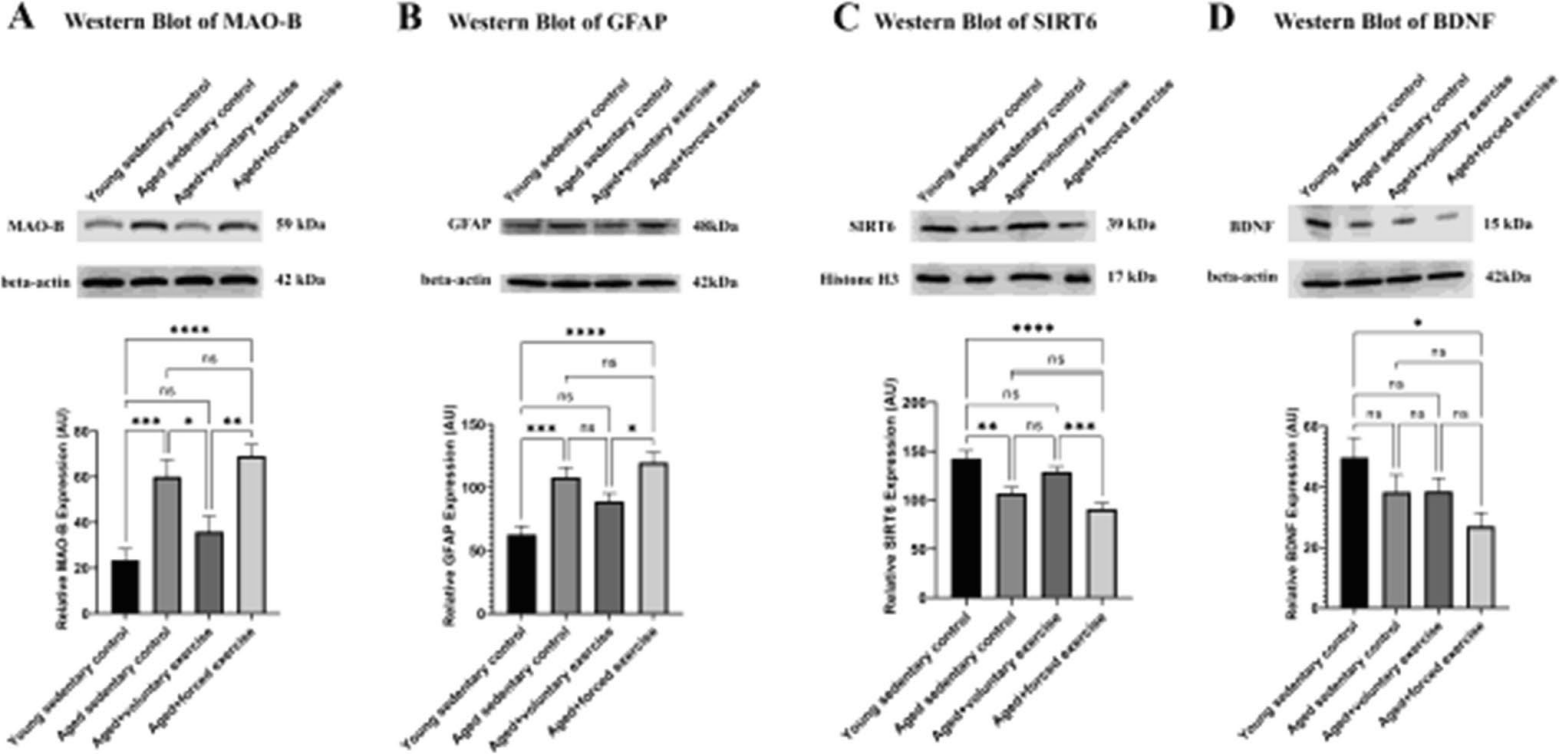
** Fig. 7** Western blot analysis of protein expression across treatment groups. This figure displays Western blot results showcasing the expression levels of crucial proteins within the retina across various experimental groups: Young Sedentary Control, Aged Sedentary Control, Aged + Voluntary Exercise, and Aged + Forced Exercise ( n = 6 in each). A The expression levels of monoamine-oxidase B (MAO-B) protein, highlighting variations in this oxidative stress marker across the groups. B The expression levels of glial fibrillary acidic protein (GFAP), an inflammatory marker. C The expression levels of Sirtuin-6 (SIRT6), signaling the activation of anti-aging mechanisms potentially influenced by physical activity. D Demonstrates the expression levels of brain-derived neurotrophic factor (BDNF), providing insights into neurotrophic support and neuronal health across the groups. Loading controls were run on separate gels. Results are expressed as mean arbitrary units (AR) with error bars representing the standard error of the mean (SEM) for clear indication of variability. Statistical significance between the groups is marked as follows: not significant (ns) for p -values greater than 0.05; * for p < 0.05; ** for p < 0.01; *** for p < 0.001; and **** for p < 0.0001

